# Event and Comment

**Published:** 1920-03

**Authors:** 


### DENTAL REGISTER

THE MAGAZINE OF DENTISTRY

Vol. LXXIV MARCH, 1920 No. 3

### EVENT AND COMMENT

A resumption in the discussions of the various principles of bridgework by their various adherents gives us some food for thought. As a preliminary suggestion a *résumé* of some of the problems of the past is interesting.

We recall the question of devitalization. Some said keep all teeth vital if possible; some said remove the pulp from every tooth used as an abutment for bridgework; while others tried to "keep a foot on each side of the fence," and said devitalize only when it interferes with the correct preparation of the abutment for the type of attachment used.

Next came the question of attachments, whether whole crown or partial crown, whether plate and dowel or band and dowel.

Then came the advocates for removable bridgework. In this the types of attachments were necessarily somewhat limited, nevertheless certain forms had their supporters and the discussions waxed warm upon the principles involved, particularly because some allowed the primary stress to be assumed by the soft tissues; others thought this should be borne by the abutments.

Then came the introduction of the casting process which gave a decided impetus to both kinds of bridgework, but more particularly to the fixed, because of the ease of construction of both dummies and partial crown attachments.

Then the X-ray brought to light our failures in root-canal technic, which seemed to indicate that more teeth should be retained vital, so naturally new form partial crown attachments and various forms of cast clasps came to the front. And so it continues with all interested supporters *ad libitum*.

Of course there has been a certain amount of progress, but the question arises in the mind of the writer with all this seeming advancement in the technical field of crown and bridgework, have we really advanced. to any great degree, have we not been going around in a circle, as a "dog chasing his tail"?

There seems to be a professional conscience which rebels to the furtherance of the cause of bridgework as practiced now. This, in the opinion of the writer, is not entirely due to the inability of the operator to carry out his ideals; nor in the thought of the harm done in extensive tooth mutilation; nor in his failure in not properly caring for devitalized teeth, but comes also from the fact that we are not serving the greatest number in the best manner.

The wholesale extraction which has been going on for the past months has greatly increased our responsibility, and this should endow us with a renewed zeal as to our moral and professional obligation to these patients.

An ideal restoration for any case should be one which requires the least number of supporting abutments, the smallest amount of tooth mutilation, the least number of teeth devitalized, and last and most important, within the means of all.

This last essential is being absolutely overlooked by most bridgeworkers. The large massive removable gold structures and the fixed structures requiring such careful technic in the construction of the partial crown attachments and their associated dummies, make them absolutely beyond the reach of the majority of people.

There are many cases where a well-planned and well-constructed plate, with the primary stress taken upon the soft tissues, and if deemed necessary, a stabilizing support upon one or more abutments, is absolutely indicated.

One plate enthusiast said a short time ago, "Well, I see the bridge advocates are now recommending a reduction in the number of abutments used in their construction. I am sure that it will be only a very short time when they will do away with all of them."

There will always be a demand for bridges, and particularly of the fixed type, because of the possibility and practicability of partial crown attachments, thereby saving the pulps of teeth. The plate, however, should come into its own, for its value from a hygienic standpoint and its comparatively low cost. The so-called removable bridge could be frequently constructed of vulcanite, instead of all gold, and thus greatly enhance its value, because of its adaptability to a greater number of patients.

---

We publish in this number an article by Thomas J. Barrett, D.D.S., of Worcester, Mass., which was read before the Pennsylvania State Dental Society and published in the *Dental Cosmos.*

It is interesting to get the viewpoint of one whom we feel belongs to the minority. The title of his paper seems to indicate to us that he has taken the wrong premise, "A New Species of Dentist." The dental hygienist never had such aspirations, nor do we believe that she can ever be persuaded to seek such an exalted position through the use of such verbose argument as presented by the essayist.

We can not see why it should be necessary to require as much time in the education of the dental hygienist as is given to the medical nurse; they are not at all comparable in any sense.

We do feel, however, that the preliminary requirements for the dental hygienist should be of a much higher standard than is now required for the medical nurse in many institutions.

The value in this paper lies in its suggestion to us of our individual duty along the line of public hygiene propaganda pertaining to preventive dentistry.

We are also publishing the first bulletin issued by "The National Anaesthesia Research Society," of which Stephen Morris, of Philadelphia, is president; B. J. Clark, Minneapolis, secretary-treasurer; and T. T. Frankenberg, of Columbus, the executive secretary.

There is also a research committee, of which F. H. McMechan, of Avon Lake, Ohio, is chairman and Wm. I. Jones, D.D.S., Columbus, Ohio, secretary.

The formation of this National Society for research in one of the branches of medicine of which we hear so little, should be of great interest to the dental practitioner, as the Society will undoubtedly give us many things of value in the future.

